# Dental Caries, Erosive Tooth Wear, and Periodontal Status of Military Student Pilots in Finland: A Comparative Cross-Sectional Study

**DOI:** 10.1093/milmed/usaf022

**Published:** 2025-01-24

**Authors:** Annakaisa Muhonen, Tarja Tanner, Jari Päkkilä, Mika Huttunen, Sari Räsänen, Pernelle Moilanen, Pertti Patinen, Tuomo Leino, Leo Tjäderhane, Vuokko Anttonen, Antti Kämppi

**Affiliations:** Finnish Defence Forces, Centre for Military Medicine, Riihimäki, 11311, Finland; Department of Oral and Maxillofacial Diseases, University of Helsinki, Helsinki, 00014, Finland; Research Unit of Population Health, University of Oulu, Oulu, 90220, Finland; North Ostrobothnia Wellbeing Services County, Oulu University Hospital, Pohde, PL 10, 90029, Finland; Research Unit of Mathematical Sciences, University of Oulu, Oulu, 90014, Finland; Finnish Defence Forces, Centre for Military Medicine, Riihimäki, 11311, Finland; Research Unit of Population Health, University of Oulu, Oulu, 90220, Finland; Finnish Defence Forces, Centre for Military Medicine, Riihimäki, 11311, Finland; Research Unit of Population Health, University of Oulu, Oulu, 90220, Finland; Research Unit of Population Health, University of Oulu, Oulu, 90220, Finland; Department of Leadership and Military Pedagogy, National Defence University Finland, Helsinki, PL 7, 00861, Finland; Department of Oral and Maxillofacial Diseases, University of Helsinki, Helsinki, 00014, Finland; Research Unit of Population Health, University of Oulu, Oulu, 90220, Finland; Department of Oral and Maxillofacial Diseases, University of Helsinki, Helsinki, 00014, Finland

## Abstract

**Introduction:**

Oral health is a crucial factor for service safety among military pilots, but studies specifically on pilots are still very few in Finland. The aim of this study was to assess the oral health status of military student pilots compared to other conscripts of the same age group.

**Materials and Methods:**

The data were collected during the oral health examinations of the annual class of the Pilot Reserve Officer Course students at the beginning of their duty at the Air Force Academy (*N* = 38). A voluntary random sample of conscripts’ oral health data (*N* = 574) collected from the 8 largest garrisons was used as a peer group. The study included males born between 2000 and 2002. The examinations were conducted in accordance with the WHO guidelines. Values of decayed (DT), missing, and filled teeth as well as the combined decayed, missing, and filled teeth value were recorded along with Basic Erosive Wear Examination scoring and Community Periodontal Index. The study was designed in accordance with the STROBE guidelines.

**Results:**

The proportion of student pilots without any decayed teeth (DT = 0) was 42.1% with third molars and 44.7% without third molars. Among other conscripts, the proportions were 29.1% and 30.8%, respectively. Mean DT values among student pilots were 1.16 (third molars included) and 1.11 (third molars not included) (*P* = .543), and among other conscripts 1.10 and 1.00 (*P* = .429), respectively.

The mean decayed, missing, and filled teeth values were 2.50 among student pilots and 3.34 among others (third molars included), and 2.18 and 3.10 (without third molars), respectively (*P* = .289 and .211).

The proportion of student pilots with at least moderate erosive tooth wear (Basic Erosive Wear Examination sum > 2) was 68.4%, while for other conscripts it was 22.1% (*P* = .000). None of the student pilots fell into Community Periodontal Index class 3 or 4, whereas 9.9% of other conscripts did (*P* = .006).

**Conclusions:**

The student pilots exhibited good overall oral health, which differed from that of other conscripts, mainly in terms of erosive changes. Continuous monitoring is crucial, as maintaining optimal oral health is vital for reducing the risk of barodontalgia and thus improving flight safety. Therefore, it is important to be aware that those who aspire to be military pilots are at an increased risk of erosive changes to their teeth.

## INTRODUCTION

In Finland, all men aged 18 to 30 years undergo mandatory military service or civilian service. For women, the military service is voluntary. In 2021, approximately 76% of men in this age group started military service, totaling 22,281 men. Women’s voluntary military service was undertaken by 1,173 women.^1^

Selections for military leadership training are typically based on the performance during service and various aptitude tests. The Pilot Reserve Officer Course at the Air Force Academy is the only leadership training program with a prearranged, highly selective multistage admission process that includes both physical and psychological sections.^2^ According to the Air Force Academy, some of the admission data of the selectees include high school grades, intelligence test value 6 or above (max 9/9), high motivation for military flying, no major medical history, and a maximal bicycle stress test of 3.45 W/kg or above. Around 40 out of hundreds of applicants are accepted annually for the course to serve their military service as student pilots. According to the Air Force Academy, there were 756 applicants in 2021. After their conscription, a small group was chosen to pursue further studies at the National Defence University with the goal of becoming fighter or helicopter pilots in the Air Force and Army or the Border Guard.

The oral health of conscripts has been a subject of systematic studies in Finland over the past 40 years. Previous studies have focused on caries prevalence and related factors as well as the temporomandibular disorders symptoms among conscripts,^3–6^ but none covered the oral health of student pilots. However, these students are a special group of conscripts and the information about their oral health status is crucial for their chosen profession.

Pressure changes related to flying or diving may cause pain in the oral cavity and dentition, which can potentially lead to an emergency during a flight or dive. This phenomenon is referred to as barodontalgia. Barotrauma is a condition in which a tooth or restoration fractures under pressure changes. Many oral pathologies might be possible sources of barodontalgia or barotrauma, including dental caries, defective restorations, pulpal necrosis, apical periodontitis, pulpitis, and impacted teeth.^7–9^ In the most unfavorable scenario, the incident could potentially result in an incapacitating event, which is incompatible with the standards of military aviation. The potential consequences could be fatal for a single-pilot military flight. To prevent these serious conditions, airworthiness requirements for military pilots include regular oral health examinations in military organizations worldwide. NATO Dental classes 1 or 2 out of scale 4 are required for a military pilot to have an aeromedical certification for military flight duty in the Finnish Defence Forces. Classes 1 or 2 mean that a dental emergency is not likely to occur within 12 months.^10^ The conscripts enrolled in the Pilot Reserve Officer Course undergo an oral health examination at the beginning of their service. In accordance with the instructions of the Defence Forces, any dental treatment deemed necessary must be completed within the first 6 months of service.

A British study by Elmer et al. suggests that Air Force recruits require less dental care than Navy and Army recruits in general. The study found that more individuals from higher social classes apply for the Air Force, while those from lower social classes are more likely to apply for the Army.^11^ The incidence of caries lesions has previously been linked to a person’s own level of education as well.^12^

The oral health of military pilots is subject to special requirements in military organizations in numerous countries. As a result, their oral health is generally better than that of non-pilot military personnel.^13^ International studies show that military pilots have lower rates of caries^12^ and periodontal disease^14^ than the general population but exhibit a relatively high prevalence of bruxism and erosive changes in the dentition.^15–17^ Military pilots access oral health care services more frequently than other soldiers, indicating a higher level of awareness and a tendency to seek emergency treatment for even minor issues.^13^ No evidence-based data on Finnish military pilots are currently available.

The aim of the study was to obtain information about the oral health of student pilots in Finland for cariological status and erosive tooth wear (ETW) as well as periodontological status. Because the oral health of the pilots is crucial, the knowledge obtained through this study is essential for providing the appropriate level of oral health services from the beginning of their career.

The hypothesis was that compared to regular conscripts in the Finnish Defence Forces, student pilots show lower caries prevalence, less ETW described as mean Basic Erosive Wear Examination (BEWE) values and lower mean Community Periodontal Index (CPI) values.

## MATERIALS AND METHODS

The research material was collected from the 8 largest garrisons in Finland during the second half of 2021 as a part of the Conscripts’ Oral Health 2021 project. A total of 10 dentists employed by the Defence Forces or performing their military service conducted examinations in the course of their daily routines. Before the study, a training and calibration session was held to ensure consistency among the dentists.

Oral health examinations were carried out at the dental clinics of the garrisons according to the WHO criteria for following the training and examination protocol explained in the previous study.^18^ The equipments used were the light and a 3-function syringe of the dental care unit, an oral mirror, a probe, and a gingival probe. The findings were recorded on the patient information system (Mediatri, Mediconsult, Finland) by a dental assistant. Bitewing radiography was not automatically part of the study protocol. Radiographs were taken only when clinically indicated, meaning the conscript was presumed to have at least one carious lesion extending into the dentin and no previous new radiographs were available.

### Calibration of Examiners

In November 2020, a training session was organized for the dentists performing the clinical examinations. During the remote session, the examination protocol, International Caries Detection and Assessment System (ICDAS) scoring,^19^ periodontal examination, and identification of the level of tooth erosion (BEWE)^20^ were defined. Following the training session, the researchers conducted a test using 55 pictures of decayed teeth (DT), which were classified according to the ICDAS system for severity/score and activity. The inter-examiner agreement was 0.66 (range: 0.38–0.76), and the intra-examiner agreement was 0.76 (range: 0.59–0.90).

### Study Group

As a part of the mandatory medical examination at the beginning of the service, a group of male conscripts born between 2000 and 2002 were offered an opportunity to participate in a voluntary oral examination. Approximately 1 in every 15 of the conscripts who began their service in July 2021 in the 8 biggest garrisons underwent an examination. These comprised the control group, reaching a total of 574 conscripts. Research subjects were selected randomly from the alphabetical list of names. A preliminary study group^21^ was included in the control group.

Student pilots are required to undergo an oral health examination to begin service, in accordance with the Finnish Defence Force regulations. The student pilot study group on the Pilot Reserve Officer Course underwent the screening at the Air Force Academy’s Military Training Squadron in Jyväskylä in July 2021 following the same guidelines. The entire study group of conscripts in the course (*n* = 38) consisted entirely of males, all born between 2000 and 2002.

Each person from both groups was asked for permission to use their dental examination data for research purposes both orally and in written form.

### Outcome Classification

Caries lesions were classified according to the ICDAS criteria.^19^ Lesions with an ICDAS score of 4 to 6 or active 3 were considered as requiring restorative treatment (DT > 0). In uncertain cases, dentists were advised to choose the more severe option. Filled (FT) and missing teeth (MT) were recorded according to WHO criteria.^22^ The examinations included the third molars. The state of eruption was not recorded on this study but was only reported as existing or missing. Erosive tooth wear was recorded using BEWE scoring.^20^ Periodontal status was recorded according to WHO criteria, as well as by using the CPI for epidemiological studies.^22^

### Ethical Considerations

The study has been authorized for trial by the Finnish Defence Forces (AQ23567) and approved by the ethical board of the University of Helsinki (no. 15/2021). All the results were pseudonymized by Statistics Finland before giving the researchers access. The material is owned by the Defence Forces and can only be accessed by the researchers mentioned in the trial authorization.

### Statistical Considerations

The mean values and standard deviations of DT, MT, FT, and decayed, missing, and filled teeth (DMFT) were calculated with and without third molars. For the cross-tabulation, DT values were categorized into 3 groups (DT = 0, DT = 1–2, and DT ≥ 3) and DMFT values into 5 groups (DMFT = 0, DMFT = 1, DMFT = 2–4, DMFT = 5–9, and DMFT ≥ 10). The BEWE sum scores were calculated according to the highest BEWE score in each sextant. These BEWE sum scores were categorized into 5 risk groups (healthy 0, mild 1–2, moderate 3–8, high 9–13, and very high 14–18).^20^ The highest CPI value in each sextant (1–4) was recorded according to the WHO criteria to analyze the periodontal state.^22^

*T*-test and 95% confidence intervals were used to compare differences in means between the groups. In cross-tabulation, differences between the groups were tested with the *χ*^2^ test. Statistical significance was determined at *P* < .05.

All the statistics were analyzed with SPSS version 28.0 (SPSS Inc., Chicago, IL, USA) and Office 365 Excel Software 2018 (Microsoft Inc., Redmond, WA, USA).

## RESULTS

Among the student pilots, the proportion of those with sound dentition (DMFT = 0) was larger than among other conscripts both including and excluding third molars. More than 60% of the conscripts in both groups had no need for restorative care. Decayed teeth < 3 covered more than 80% of both student pilots and other conscripts. The mean DMFT value among student pilots was lower than other conscripts, both with and without third molars (*P* = .289 and 0.211, respectively). Student pilots had a slightly higher mean DT value (*P* = .542/.429). When third molars were included, the mean MT was slightly higher among student pilots (*P* = .260). The mean FT value was lower among student pilots than other conscripts, regardless of including or excluding third molars (*P* = .018 and .018, respectively) (**Table 1**).

**Table 1.** Proportions of Individuals in Different DMFT and DT Value Categories in the Student Pilots and Other Conscripts’ Groups and Mean DMFT, DT, MT, and FT Values with Standard Deviations (SD), *P*-values and 95% confidence intervals (95% CI) for the difference between means. a: third molars included; b: third molars excluded.

a	Student pilot	Other		
DMFT	% (*n*)	% (*n*)		
0	42.1 (16)	29.1 (167)		
1	10.5 (4)	14.8 (85)		
2–4	26.3 (10)	2.8 (154)		
5–9	15.8 (6)	22.0 (126)		
10–32	5.3 (2)	7.3 (42)		
Total	100 (38)	100 (574)		
DT				
0	65.8 (25)	62.9 (361)		
1–2	15.8 (6)	20.2 (116)		
3–32	18.4 (7)	16.9 (97)		
Total	100 (38)	100 (574)		
	Mean (SD)	Mean (SD)	*P*-value	95% CI
DMFT	2.50 (3.33)	3.34 (3.81)	.289	−0.41, 2.09
DT	1.16 (2.11)	1.10 (2.05)	.543	−0.73, 0.61
MT	0.26 (0.72)	0.19 (0.70)	.260	−0.31, 0.16
FT	1.11 (1.74)	2.05 (2.60)	.018	0.11, 1.79
**b**	Student pilot	Other		
DMFT	% (*n*)	% (*n*)		
0	44.7 (17)	30.8 (177)		
1	13.2 (5)	15.5 (89)		
2–4	26.3 (10)	27.0 (155		
5–9	10.5 (4)	20.2 (116)		
10–28	5.3 (2)	6.4 (37)		
Total	100 (38)	100 (574)		
DT				
0	65.8 (25)	64.3 (369)		
1–2	21.1 (8)	20.7 (119)		
3–28	13.2 (5)	15.0 (86)		
Total	100 (38)	100 (574)		
	Mean (SD)	Mean (SD)	*P*-value	95% CI
DMFT	2.18 (3.13)	3.10 (3.66)	.211	−0.28, 2.11
DT	1.11 (2.08)	1.00 (1.94)	.429	−0.75, 0.54
MT	0.00 (0.00)	0.05 (0.36)	.074	−0.06, 0.17
FT	1.11 (1.74)	2.05 (2.59)	.018	0.11, 1.78

Abbreviations: DMFT, decayed, missing, and filled tooth; DT, decayed tooth; FT, filled tooth; MT, missing tooth.

More than 40% of other conscripts showed no signs of ETW (BEWE = 0), while the same was true for 8% of the student pilots. Those with moderate or severe ETW (BEWE sum score 3–13) comprised nearly 70% of student pilots and slightly more than 20% of other conscripts. The difference between the groups was statistically significant (*P* < .01) (**Table 2**).

**Table 2.** Distributions of Basic Erosive Wear Examination (BEWE) sum score values in Pilot Reserve Officer Course student pilots and other conscripts’ groups. *P* < .01.

BEWE	0	1–2	3–8	9–13	Total
% (*n*)					
Student pilot	7.9 (3)	23.7 (9)	65.8 (25)	2.6 (1)	100 (38)
Other	43.7 (247)	34.2 (193)	21.4 (121)	0.7 (4)	100 (565)

A CPI value of 0 was not observed among student pilots, while more than 8% of other conscripts had CPI = 0. The student pilots were not found to have CPI values higher than 2 either. In contrast, among other conscripts, CPI values of 3 to 4 comprised a total of 10% of the group. The difference between the groups was statistically significant (*P* = .006) (**Table 3**).

**Table 3.** Distributions of highest Community Periodontal Index (CPI) score values in Pilot Reserve Officer Course student pilots and other conscripts’ groups. *P* = .006.

CPI	0	1	2	3	4	Total
% (*n*)						
Student pilot	0.0 (0)	28.9 (11)	71.1 (27)	0.0 (0)	0.0 (0)	100 (38)
Other	8.7 (49)	38.8 (219)	42.6 (240)	8.5 (48)	1.4% (8)	100 (565)

## DISCUSSION

The Pilot Reserve Officer Course did not appear to differ from other conscripts in need of restorative caries treatment (DT). However, the overall oral health (DMFT, CPI) of student pilots was better than that of other conscripts, which concurs with the hypothesis. Previous restorative caries treatment (FT) was statistically significantly lower among them compared to other conscripts. On the other hand, student pilots had significantly more ETW than other conscripts, which clearly contradicts our hypothesis. They also had slightly more third molars removed or missing before conscription.

In comparing the situation with other conscripts, it is noteworthy that 25% of each age group is exempt from conscription, having chosen civilian service or based on health grounds. Therefore, it is probable that the least favorable segment within the age group was absent from this study. The size of study group was relatively limited as well, which precludes interpreting the results through individual figures and their statistical significance alone; rather, the results should be viewed as indicative. A previous study on Finnish conscripts by Huttunen et al. found that individuals who regularly engaged in sports require less restorative caries treatment than those who did not participate. Additionally, good physical fitness was negatively associated with the need for restorative caries treatment.^23^ Young people who engage in physical activity have also been found to have better oral hygiene and eating habits than those who are not active in sports.^24^ Oral health factors can also impact physical condition.^25^ Excellent physical condition is one of the most important qualities of a military pilot, and student pilots are among the best in their age group, both mentally and physically, based on the admission requirements. The study also shows less caries history and previous need for dental treatment among them.

In Finland, everyone under the age of 18 years is entitled to free public dental care. All minors have regular statutory dental check-ups at the same age. Compared to student pilots, other conscripts had more diagnosed and treated caries before military service. However, both groups had equal mean levels of need for restorative caries treatment at the time of our study. This discrepancy may be because of the student pilots being classified as having a lower caries risk, which resulted in a reduction in the provision of preventive treatment compared to the control group and, additionally, may have delayed manifestation of caries lesions. In previous conscript data, only a small proportion of conscripts were shown to carry the caries burden as only 30% of conscripts had 90% of all DT.^5^ This is in line with the findings in our study, as few as 15% of the participants had 3 or more caries lesions.

The difference in ETW between the groups was significant, as two-thirds of the student pilot group had moderate or high tooth wear (BEWE sum score 3–13), whereas the same was true only for less than a quarter of other conscripts. The global prevalence of dental erosion is between 20% and 45%.^26^ Lim et al. reported that 22% of young soldiers aged 18 to 25 years in Singapore showed erosive changes in their teeth.^27^ These figures are lower than in our study. This is an important finding given the future work profile and circumstances of military pilot students. Erosive tooth wear can eventually lead to severe loss of tooth substance and pulpal infections, which require rehabilitation of the entire dentition. Tooth wear is an ambiguous phenomenon and, especially, in the first phase, it is difficult to differentiate between erosion, attrition, and abrasion. In practice, they often overlap and occur simultaneously.^28^ Differentiating between attrition, abrasion, and erosion requires more detailed background information and is a subject of further research.

However, occlusal surface wear of dentition is related to bruxism. Among military pilots, up to 70.6% have been found to have changes in dentition that are related to bruxism.^15^ The prevalence of bruxism in the general adult population is reported to be 8% to 31.4%.^29^ An association between occupational stress and bruxism among military pilots has been demonstrated in several studies.^15,16,30^ The Pilot Reserve Officer Course psychological aptitude test indicates that student pilots are a highly conscientious group. Furthermore, they generally outperform their peers at school, which suggests that they may have experienced greater academic pressure before enlistment. This may partly explain the high proportion of those individuals with at least moderate ETW in this study. Given that they exhibit more erosive changes than the general population, even before becoming pilots, it is imperative to continue investigating both preventive measures and occupational health and safety measures to prevent the problem from worsening during their careers.

The Community Periodontal Index is not an ideal way to determine the periodontal status at the individual level, but it is useful in epidemiological research to obtain information on a larger number of people.^22^ Prevalence of severe periodontitis has been found to be around 10% in every population, and it can already start in adolescence.^31^ Mean CPI 3 or 4 among other conscripts corresponds to this. The mechanism of disease development is very complex and is influenced by a number of factors, such as genetics and systemic diseases.^32^ Many congenital conditions that predispose one to severe periodontitis are also a reason for exemption from military service. In the case of young people, deepened gingival pockets could indicate the state formerly known as aggressive periodontitis. However, previous research shows that it is not a very common problem in Finland and Northern Europe, prevalence being around 0.1%.^33^ However, many risk factors for periodontitis are directly linked to a person’s life management^34^ and thus the lower CPI scores observed in the pilot student group compared with other conscripts partly contribute to their healthier lifestyle. The smaller size of the research team may also affect the result. Thankappan et al. reported that only ∼8% of military aviation crew suffer from active periodontitis, whereas the Health 2000 study found that ∼64% of the adult Finnish population has deepened gingival pockets.^14,35^ Therefore, while periodontitis may not be prevalent among pilots, as was also the case in the study population, it is important to acknowledge the potential impact of stressors on inflammatory conditions in the body.

Along with the stressful occupational environment, barodontalgia is a phenomenon potentially affecting military pilots’ dentition during their careers and avoiding it is the main focus of military oral health care. It is thus important to know the baseline for the measures required to reduce the risk of barodontalgia as close to zero as possible. It is also noteworthy that dental barotrauma of a sound tooth has been described in the literature.^36^ The majority of student pilots do not require dental treatment. One-third of the 38 student pilots required one or more dentist appointments for fulfilling the Air Force requirements for airworthiness. Half of those needing caries treatment had 1 to 2 DT. Therefore, the requirement for all the treatment being done during the first 6 months of the service can be met in most cases. Those with the highest caries activity and ETW should be targeted for improved self-care instruction. It is also of the utmost importance for all the student pilots to be aware of the risks associated with barodontalgia, as well as the potential impact of bruxism and stress factors.

A slightly higher number of extracted third molars among student pilots are a noteworthy finding in light of the fact that many of the pathological conditions causing barodontalgia manifest themselves in the third molar region.^8^ Partially erupted third molars are also one of the main reasons for hospitalization during deployment.^37,38^ Evacuation to a hospital with surgical facilities is time-consuming and, in the case of a fighter pilot, especially expensive. Even during conscription, third molar extraction causes a loss of effective service time. The stage of eruption and position as well as the reason for missing third molars is an important topic of future research.

However, the greater number of extracted third molars may be linked to the higher socioeconomic status of student pilots. Despite the fact that Finnish children are entitled to equal dental care until the age of 18 years, a significant proportion of patients subsequently seek treatment at private clinics, where it may be more straightforward to have partially erupted third molars extracted preventively compared to public health care where the preferred course of treatment is often more conservative. Student pilots might also be more aware of the impact that partially erupted third molars could have on their military service, and thus may visit the dentist for less serious dental issues.

The clinical examinations took place during the peak of the COVID-19 pandemic, and strict guidelines from the Ministry of Social Affairs and Health were followed. The Finnish Defence Forces also had their own restrictions, which made the inspections time-consuming. One examination lasted ∼approximately 15 minutes, which is considerably longer than in the previous study.^18^ Delays and cancellations occurred because of the quarantine or isolation of either certain groups of conscripts or dentists conducting examinations. As a result, considerably fewer inspections were carried out compared to previous studies.^3–6^ Nevertheless, the sample is representative, and, despite the restrictions, the quality of the sample was not compromised; neither did the pandemic influence the results of the study.

The number of participants varies slightly according to the variable (DT, CPI, and BEWE). This discrepancy is likely because of a typographical error or the omission of a requisite numerical value within the designated field on the patient information system. However, the variation is limited.

The major limitations of the study are the COVID-19 pandemic, which also set some limitations to the study design and the small sample size. Areas for future study are the background factors of the ETW among student pilots as well as the status of third molars.

## CONCLUSIONS

Military pilots are known to have more ETW than the regular population, which our study shows among student pilots as well. The Pilot Reserve Officer Course conscripts have less history of caries treatment and better periodontal status in comparison with the other conscripts. This correlation is consistent with what is known about their background as conscientious and sporting individuals. However, their caries treatment requirements do not differ from other conscripts.

The results confirm the need for continuous monitoring of the oral health of the student pilots and targeting resources to prevent ETW. However, further research is required to identify the underlying factors contributing to ETW, to target preventive measures for those at higher caries risk and also the stage of eruption and position of third molars.

## Data Availability

The material is owned by the Defence Forces and can only be accessed by the researchers mentioned in the trial authorization.

## INSTITUTIONAL CLEARANCE

Institutional clearance does not apply.
